# Effect of authentic leadership on work readiness: the mediating role of nurses’ agility

**DOI:** 10.1186/s12912-024-02362-5

**Published:** 2024-10-11

**Authors:** Ahmed Farghaly Tawfik, Shimaa Abd El-fattah Mahgoub

**Affiliations:** Lecturer of Nursing Administration, Beni-Suef University, Beni-Suef, Egypt

**Keywords:** Authentic leadership, Work readiness, Agility, Nurses, Mediation

## Abstract

**Background:**

The dynamic and demanding nature of healthcare environments necessitates that nurses not only possess clinical proficiency but also exhibit high levels of work readiness to adapt swiftly to changes. Authentic leadership has been recognized as a critical factor influencing various organizational outcomes.

**Aim:**

Investigating the mediating role of nurses’ agility in the relation between authentic leadership and nurses’ work readiness.

**Design and method:**

A correlational analytical research design was utilized following STROBE guidelines, and data were collected from 249 nurses at a hospital affiliated with Beni-Suef University, Egypt. Instruments included authentic leadership Questionnaire, work readiness scale, and workforce agility scale. Data was collected from the beginning of March to the end of April 2024.

**Results:**

The findings indicate that authentic leadership was notably strong regarding morality/ethics dimension (mean score: 15.81), and nurses demonstrated relatively high agility levels, particularly in proactivity (mean score: 29.16). Organizational acumen scored highest in work readiness dimensions (mean score: 53.94). Nurses’ overall scores for study variables ranged from 72 to 80% of the maximum, with agility scoring highest (mean score: 85.77). Significant positive correlations were found between variables, especially between nurses’ agility and authentic leadership (*r* = 0.362).

**Conclusion:**

Path analysis reveals nurses’ agility as a paramount mediator between authentic leadership and nurses’ work readiness, indicating its vital role in transmitting the positive effects of authentic leadership. Practical implications include establishing authentic leadership programs that foster nurses’ agility especially proactive behaviors. That in turn improve nurses’ readiness for various work responsibilities.

## Background

Nursing work force composes a large and vital partition of the healthcare workforce. According to WHO (2024), there are an estimated 29 million nurses worldwide and 2.2 million midwives. WHO predicts a shortage of 4.5 million nurses and 0.31 million midwives by 2030. In a competitive and fast-dynamic world, a competent and committed workforce represents a nuclear weapon in a war that helps organizations to survive and push toward the future. Affective and competent leadership and management of nurses result in improved quality of health care. Authenticity, a vital concept associated with positive interactions and cohesiveness within group dynamics, is essential for the effective performance of individuals and organizations [1].

In the recent work environment where competition among employees increased with different mindsets and various cultural and ethical backgrounds besides, increased stress, employees hardly build a trustful relation with colleagues and co-workers [2]. To restore integrity, hope, and confidence, organizations need to switch from charismatic leadership to value-based practices as authentic leadership [3].

The main concept of authenticity is to accept, know, and remain consistent with oneself. Authentic leaders know in depth about their behaviors and are very cautious about their own and other perceived morals, values, beliefs, understanding, and strengths [4]. According to a systematic review of reviews by [5] there is plenty of data illustrating the advantages of value-based nursing leadership styles “authentic leadership” for outcomes for nurses, organizations, and patients. Authentic leadership is a new value-based style that supports healthy work environments, assists nurses in discovering their job purpose, and fosters transparent interactions that boost optimism and trust [6]. Authentic leaders as role models foster nurses’ work readiness [7].

Nurses’ work readiness refers to their preparedness to enter and excel in the workforce. It encompasses a combination of knowledge, skills, attitudes, and personal attributes that contribute to successful performance in their roles. Authentic leadership can positively influence nurses’ work readiness by creating a supportive and transparent work environment, fostering self-awareness and continuous development, and promoting a positive and resilient culture within the healthcare setting [8].

Work readiness does not independently occur but is influenced by interrelated factors [9]. Work environment has a set of conditions or factors that either interfere with work readiness at an individual level [10]. Many personal, educational, and community factors interact in diverse ways to influence nursing students’ readiness to practice [7]; confidence in clinical practice [11, 12], and fluidity with resiliency [13]. Ethical and professional behaviours are essential for nurses, as they must adhere to ethical standards and maintain a high level of professionalism [14]. When combined, nursing students who lack the necessary noncognitive skills, including resilience as a major dimension of agility combined with adaptability and proactivity, may feel more anxious, stressed out, and mentally ill, which in turn lessens nurses work readiness [15].

Workforce agility is conceptualized as the nurses’ ability to adapt to unexpected changes; to operate effectively under stressful conditions in a dynamic environment, and to adapt to new work demands [16]. The concept of nurses’ agility as outlined by [17] refers to the behavior of nurses who demonstrate the ability to act proactively, be adaptable, and show resilience. Workforce agility has a positive correlation with task performance, innovative performance, organizational citizenship behavior, satisfaction, and well-being [18]. When faced with setbacks, resilient people used to see the situation positively. It would be interesting to connect the aforementioned workforce agility dimensions given their nature [19].

### Theoretical framework

Identifying and arranging important ideas, connections, and variables in a way that offers a well-organized framework for the study is the process of developing a theoretical framework. Reviewing existing literature provide insights into the following relationships.

Authentic leadership theory, developed by [20], and social cognitive theory developed by [21] can be utilized to interpret theoretical connections among authentic leadership, agility, and nurses’ work readiness. While authentic leadership is crucial, it alone is insufficient for achieving desired goals [20]. Authentic leadership operates by illustrating how such leaders can influence followers’ attitudes and behaviors. Authentic leadership theory is posited to the idea of leading by example/role modeling through setting high moral standards [22].

Social cognitive theory emphasizes the role of observational learning, confidence, and personal abilities in shaping behaviours and outcomes. Authentic leadership can be viewed a factor influencing nurses’ perceptions of their work environment and their own behaviors [23], agility acts as an intermediary between authentic leadership and nurses’ readiness for work, indicating that how nurses perceive and react to leadership influences their readiness [24].

Most existing literature focuses on enhancing nurses’ readiness through training programs or preceptorship programs [25–28]. Maybe it’s time to reframe the question to ask whether the workplace is ready to support newly graduated nurses, rather than whether they are sufficiently prepared for it [29]. Here, authentic leadership may be a promising strategy to support the transition to the workplace, strengthening confidence in nurses’ own ability to cope [30, 31].

**Fig. 1:**
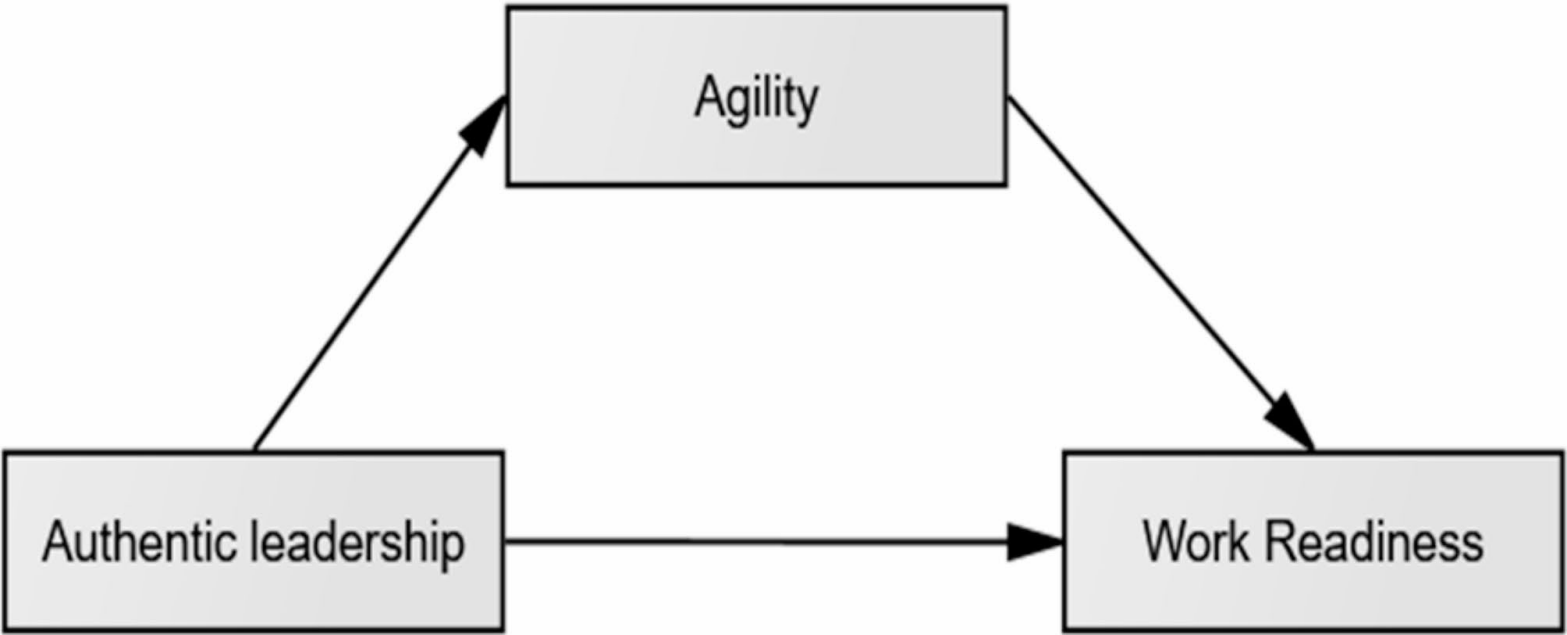
Theoretical framework for relation between authentic leadership, nurses’ agility, and work readiness

Moreover, value-based leadership positively influences employee agility; authentic leadership [32, 33], transformational leadership [34], humble leadership [35], empowering leadership [36] [37]. found that the mechanism through which leaders stimulate followers’ behavior has not been well analyzed. Further research is still needed to substantiate these claims, according to [38], to confirm the AL-behavior linkage needed to identify the relevant mediating factors. agility used in previous studies [34] as a mediator between transformation leadership and employee work-related outcomes such as work creativity and psychological safety. Nurses’ agility, in turn, impacts work readiness by influencing the nurses’ ability to navigate changes, solve problems, and acquire new skills. Resilience may be an indicator of practice readiness [39]. Based on prior literature, the following theoretical framework is conceptualized as illustrated in Fig. 1. this model was later modified based on the statistical insignificance of the direct effect of authentic leadership on work readiness; Table 5.

This can be justified in the light of authentic leadership theory which mentioned, and I quote “although authentic leadership is important, it is not sufficient to achieve desired goals” [20]. In our study context, it could be said that nurses need energizing power that transmits the positive effects of authentic leaders to produce the desired outcome of “work readiness”.

### Significance of the study

Having strong leadership is essential to building agile organizations. It is noteworthy that responsible leadership fosters learning agility, adaptability, and a greater degree of stakeholder orientation, all of which have an impact on workforce agility [40]. The study addresses a significant gap in the literature by examining how authentic leadership as a value-based leadership can optimize nurses’ readiness and agility role as a mediator that emphasizes the importance of nurses’ personal characteristics, offering valuable insights for supporting the successful integration of newly graduated nurses into healthcare settings [41]. This research is particularly timely as it addresses current challenges and opportunities in healthcare leadership and workforce dynamics, aiming to enhance organizational effectiveness and nurse satisfaction in delivering high-quality patient care.

### Aim of the study

The aim of the study was to investigate the mediating effect of nurses’ agility in the relation between authentic leadership and nurses’ work readiness.

### Research question

Does nurses’ agility play a mediating effect in the relation between authentic leadership and nurses’ work readiness?

## Subjects and methods

### Research design

A correlational analytical research design was utilized to carry out this study following STROBE guidelines.

### Study setting

The study was conducted at a hospital affiliated to Beni-Suef University, Beni-Suef governorate, Egypt. This facility consisted of two main buildings with six floor and provides non-paid health services to a wide scale of populations in lower Egypt. It is equipped with a wide variety of departments and units that cover almost all the common and rare medical and surgical specialties.

### Subjects

A convenience sample from nurses (249) out of (975) to the following inclusion criteria.

#### Inclusion criteria

Nursing staff (a) working at the previously mentioned study setting, (b) who were available during the data collection period and agreed to participate in the study, (c) with minimum experience of one year (d) those who provided informed consent for voluntary participation. The population was documented as 975 nurses, with a margin of error of 5 units and a significance threshold (p) set at *p* ≤ 0.05. To perform a SEM analysis, a recommended minimum sample size of 200 was established, meeting [42] criteria of at least 200 samples to ensure statistical relevance. Consequently, To achieve this, a questionnaire was administered conveniently to 300 nurses. 249 nurses completed and returned the surveys, forming the designated target sample.

### Tools of data collection

Data collection of this study achieved using three tools: Authentic Leadership Questionnaire, Work readiness scale, Workforce agility Scale.

#### **Tool I:-** authentic leadership questionnaire

It consists of two parts; **part I**: socio-demographic characteristic: This part developed to collect data about socio demographic characteristics of study subjects including: age, experience, gender, material status and previous education. **part II**: Authentic Leadership Questionnaire. This tool was developed by [43] and adopted from [44]. to gauge nurses’ perceptions of hospital administration authentic leadership applied at practice. This questionnaire was consisted of 16 items, divided into four dimensions named: transparency, morality, Balanced Processing and Self-Awareness. The scale on the questionnaire had four possible answers: zero meant “not at all,” one meant “once in a while,” two meant “sometimes,” three meant “fairly often,” and four meant “frequently, if not always.” the internal consistency reliability was 0. 956 (Cronbach’s Alpha).

#### **Tool II:-** work readiness scale

This questionnaire was used to assess nurse-interns’ work readiness characteristics developed by [45] and adopted from [46]. is a four dimensions questionnaire with (43 items) measured on a 5-point Likert scale. Four dimensions namely: work competence (13 items), social intelligence (9 items), organizational acumen (14 items), personal work characteristics (7 items) the internal consistency reliability was 0. 989 (Cronbach’s Alpha).

#### **Tool III:-** workforce agility scale

This scale was developed by [47] and modified by [48]. This scale, which has (21 items) divided into three categories—Proactive (seven things), Adaptive (seven items), and Flexible (seven items)—was designed to gauge the degree of workforce agility among nurses. A five-point Likert scale, with 1 denoting strongly disagree and 5 denoting strongly agree, was used to rate the subjects’ answers. High score indicates higher nurses’ agility and vice versa. the internal consistency reliability was 0. 868 (Cronbach’s Alpha).

### Validity of the tools

The translation and validation process of the study tools involved multiple steps to ensure accuracy and credibility [49]. Initially, two professional translators independently translated the study tools from English to Arabic, focusing on maintaining the original meaning and context. Following this, two additional independent translators conducted a back-translation from Arabic to English to identify any discrepancies or loss of meaning. The accuracy and validity of the Arabic translation were then evaluated by a panel of five experts, including professors from the nursing administration department. This panel assessed content and face validity, examining the translation’s relevance, clarity, completeness, appearance, and appropriateness. and some words were adjusted to fit the Egyptian context. Each item was rated on a scale of 1 (not necessary) to 3 (essential), and the CVI was calculated for each item. Items with a CVI of 0.78 or higher were retained, indicating a high level of agreement among the experts about the relevance of the items.

## Methods

The study was executed according to the following steps:

### Approval

Before starting on the study, official approval was issued from the manager of Beni-Suef University hospital to facilitate data collection. Individual written consent was also obtained from each participant in the study.

### Preparatory phase

This phase conducted in December 2023 to January 2024. In order to become familiar with the topic, the researcher studied the relevant literature from the past and present during this phase. Additionally, local and global literature and knowledge relevant to the study are incorporated into the tools for data gathering by means of books, articles, magazines, and the internet. The study employed valid tools.

### Pilot study

Prior to beginning fieldwork and data collecting, a pilot study including 10% of the primary study participants (25 staff nurses) was carried out. A pilot research was conducted to evaluate the study’s relevance and the clarity of the questionnaire sheets. Estimating the amount of time required to finish the data gathering forms was also helpful. Filling up the forms took ten to fifteen minutes. The pilot research participants were not included in the primary study population.

### Field work

For two months, the study’s fieldwork was conducted. In 2024, data gathering was place between March and April. The researchers met with the faculty members and gave them an explanation of the study’s aims after receiving formal approval to conduct the investigation. Faculty and staff members were contacted during working hours based on their availability for three days a week; the daily count of staff members interviewed varied between eight and twelve.

### Ethical consideration

The **Beni-Suef University Faculty of Medicine’s Scientific Research Ethical Committee** granted ethical approval before the commencement of the research investigation. Approval code **FMBSUREC/07072024/Tawfik** The participants were advised that the information gathered would be kept private and that they might withdraw at any moment and for any reason.

### Statistical design

Before being entered into the computer, data were verified. The Statistical Package for Social Sciences created by IBM, Illinois, Chicago, USA (SPSS version 25.0) was used for that purpose, followed by data analysis and tabulation. Descriptive statistics were employed to present the characteristics of both study participants and variables The mean scores were computed for numerical values. When *p* ≤ 0.00 L, a highly significant level value was taken into consideration, and the level of significance was selected at *p* < 0.05. For mediation model created using SPSS-AMOS V. 26. Parametric tests were used as the data showed normal distribution according to the Kolmogorov- Siminouv test. Variables’ multicollinearity was assessed, confirmed tolerance was > 0.1, and the variance inflation factor (VIF) was < 3, showing no multicollinearity. Pearson’s correlation was used to analyse bivariate correlations between the study variables.

### Preliminary analysis

The hypothesised model was assessed using AMOS’s structural equation modelling (SEM). The confirmatory factor analyses (CFA) findings indicated that the model fit the data well. The tests applied in this study were the chi-square (χ2) test of model fit, with a ratio of χ2/df < 5 representing a good model fit. Other tests used were the comparative fit index (CFI ≥ 0.95), Tucker–Lewis index (TLI ≥ 0.95), and root mean square error of approximation (RMSEA < 0.08) [50].

## Results

Table 1 illustrates staff nurses personal characteristics, the highest age category reported was between 25 to > 35 (49%) with mean age of 25.85, the highest category of experience reported between 5 to less than 10 years (46.6%), female gender was the predominated gender with (62.7%). Approximately half staff nurses were un-married (52.2%) and with diploma qualification (53.0%).

**Table 1:** Distribution of staff nurse according to their personal characteristics (*N* = 249)

Personal characteristics	Academic	
	N	%
Age groups:		
• < 25	113	45.4
• 25-	122	49
• 35-	11	4.4
• 45+	3	1.2
Mean + SD	25.85 + 4.76	
Years of experience in nursing: -		
• 1-	80	32.1
• 5-	116	46.6
• 10-	42	16.9
• 15+	11	4.4
Gender		
• Male	93	37.3
• Female	156	62.7
Marital status		
• Un-married	130	52.2
• Married	119	47.8
Qualifications		
• Diploma	132	53.0
• Bachelor	93	37.3
• Master	24	9.7
Total	249	100

Table 2: Displays means and standard deviation of nurses’ scores regarding study variables dimensions, the highest Authentic leadership dimensions mean score was Morality/Ethics (15.81), and the highest Agility dimensions mean score was proactivity (29.16). concerning work readiness dimensions highest dimension mean score was organizational acumen (53.94).

**Table 2:** Mean and standard deviation of authentic leadership, agility and work readiness dimensions among staff nurses (*N* = 249)

Study Variables Dimensions	Max Score	Academic	
		Mean ± SD	
**Authentic leadership dimensions**			
Transparency	25	19.41	3.941
Morality/Ethics	20	15.81	2.972
Balanced processing	15	11.68	2.459
Self-awareness	20	15.46	3.326
**Agility dimensions**			
Proactivity	35	29.16	3.16
Adaptability	35	28.59	3.309
Resilience	35	28.01	3.345
**Work readiness dimensions**			
Work Competence	65	49.29	4.39
Social Intelligence	45	33.53	2.99
Organizational Acumen (OA)	70	53.94	4.17
Personal Work Characteristics (PWC)	35	20.90	5.60

Table 3 illustrates that staff nurses’ mean scores relatively high as mean scores ranged from 72 to 80% of max. score for all study variables and the highest mean score was for nurses’ agility (85.77).

**Table 3:** Mean and standard deviation of study variables staff nurses (*N* = 249)

Psychological ownership dimensions	Max Score	Administrative	
		Mean ± SD	
Nurses’ agility	105	85.77	7.468
Authentic leadership	80	62.36	11.318
Work readiness	215	156.66	10.33

Table 4 shows the presence of significant positive correlations between study variables strongest correlation was between nurses’ agility and authentic leadership (*r* = 0.362), while weakest correlation was between work readiness and authentic leadership (*r* = 0.136).

**Table 4:** Correlation matrix of study variables

Pearson correlation coefficient				
	1	2	3	4
1. Workforce agility				
2. Authentic Leadership	0.362**			
3. Work readiness	0.171**	0.136*		

**Fig. 2:**
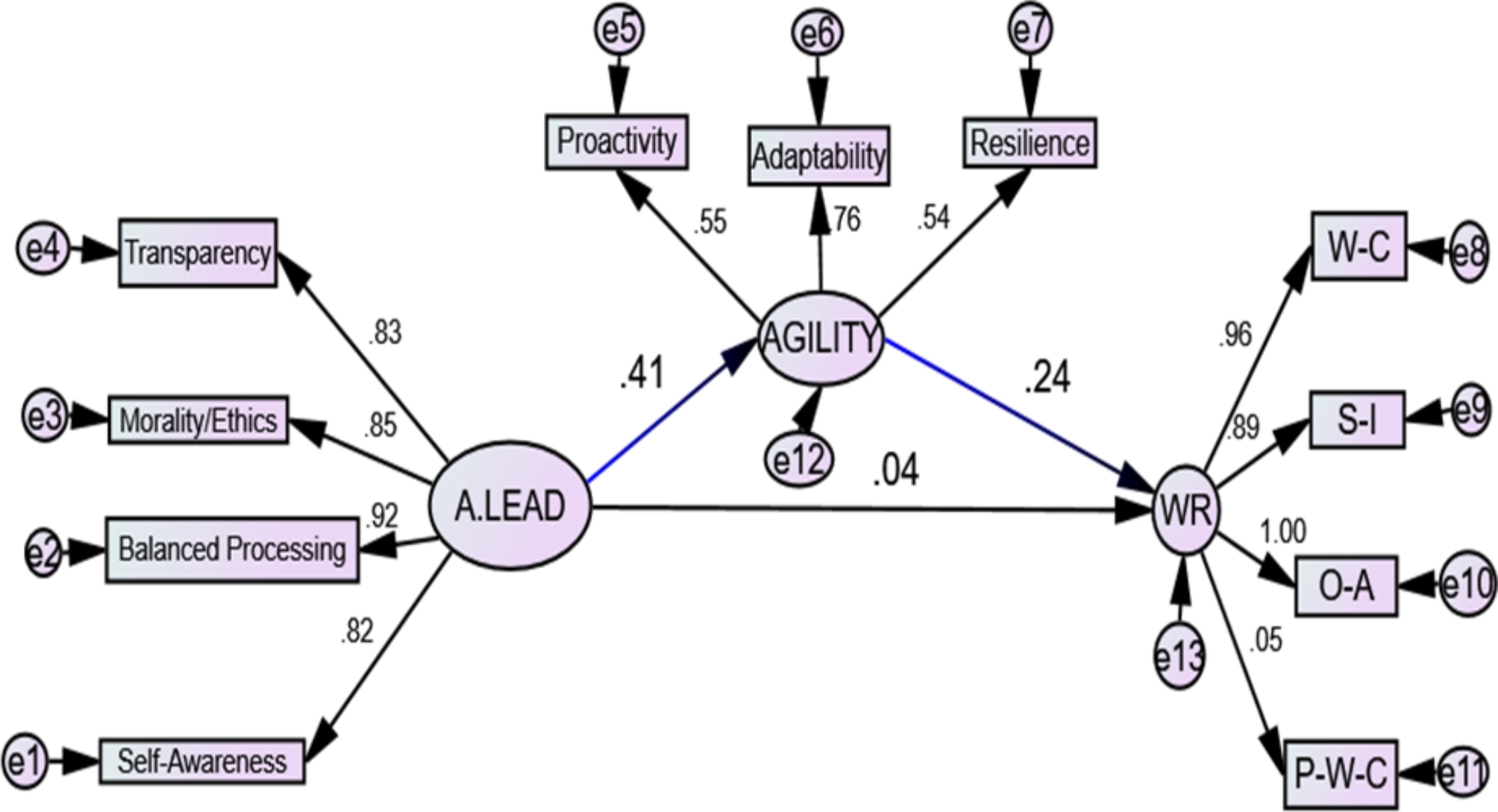
Path analysis of direct and indirect effects of authentic leadership on work readiness mediated by agility.(A.Lead = authentic leadership, W-C = work competence, S-I = social intelligence, O-A = organizational acumen, P-W_C = personal work characteristics, and WR = work readiness)

Figure 2. and Table 5: present the results from the analysis, showcasing standardized regression weights, standard errors (SE), critical ratios (CR), and significance (p-values) of only indirect effects of authentic leadership on nurses’ work readiness; as the direct effect of authentic leadership on nurses’ work readiness was insignificant. These effects are mediated through the construct of nurses’ agility. The statistical analysis was conducted using SPSS-AMOS. The study’s variables produced reliable estimates, with the comparative fit index indicating a high model fit and the root mean square approximation error also indicating a good fit. The model fit parameters met satisfactory standards (CMIN/DF = 2.491, GFI = 0.934, CFI = 0.968, RMSEA = 0.078). This model was modified because of the direct effect of authentic leadership on work readiness not statistically significant (*p* = 0.574).

**Table 5:** A path analysis of direct and indirect effects of authentic leadership on work readiness mediated by agility

Variable 1	Direction	Variable2	Standardized Regression Weights	S.E.	C.*R*.	*P*	Significance
AGILITY	<---	A.LEAD	0.261	0.059	4.402	***	Significant
WR	<---	AGILITY	0.993	0.380	2.616	0.009	Significant
WR	<---	A.LEAD	0.113	0.201	0.562	0.574	Not Significant
Self Awareness	<---	A.LEAD	1.000				Significant
Balanced Processing	<---	A.LEAD	0.832	0.047	17.524	***	Significant
Morality/Ethics	<---	A.LEAD	0.935	0.059	15.926	***	Significant
Transparency	<---	A.LEAD	1.208	0.079	15.321	***	Significant
Proactivity	<---	AGILITY	1.000				Significant
Adaptability	<---	AGILITY	1.466	0.253	5.795	***	Significant
Resiliency	<---	AGILITY	1.046	0.184	5.669	***	Significant
Work Competence	<---	WR	1.000				Significant
Social Intelligence	<---	WR	1.103	0.041	26.691	***	Significant
Organizational Acumen	<---	WR	1.568	0.032	48.646	***	Significant
Personal Work Characteristics	<---	WR	0.035	0.042	0.829	0.407	Significant

Model fit parameters; CMIN/DF = 2.491, GFI = 0.934, CFI = 0.968, RMSEA = 0.078. Chi-square = 102.140, Degrees of freedom = 41, Probability level = 0.000. CFI: Comparative Fit Index, IFI: Incremental Fit Index, RMSEA: Root Mean Square Error of Approximation.

A.LEAD= authentic leadership, WR = work readiness

**Fig. 3:**
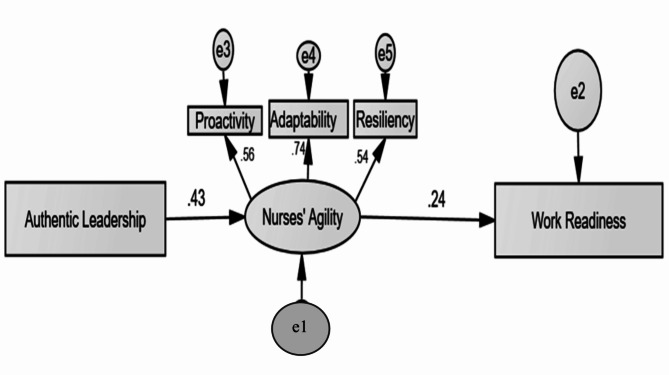
Modified path analysis of indirect effects of authentic leadership on work readiness mediated by agility

Figure 3. and Table 6: present the results from the modified path analysis, showcasing standardized regression weights, standard errors (SE), critical ratios (CR), and significance (p-values) of both the indirect effects of authentic leadership on nurses’ work readiness. These effects are fully mediated through the construct of nurses’ agility. The statistical analysis was conducted using SPSS-AMOS. The study’s variables produced reliable estimates, with the comparative fit index indicating a high model fit and the root mean square approximation error also indicating a good fit. The model fit parameters met satisfactory standards (CMIN/DF = 2.263, GFI = 0.98, CFI = 0. 956, RMSEA = 0.071).

**Table 6:** Modified path analysis of direct and indirect effects of authentic leadership on work readiness mediated by agility

Variable 1	Direction	Variable2	Estimate	S.E.	C.*R*.	*P*	Significance
Agility	<---	Authentic Leadership	0.068	0.014	4.922	***	significant
Proactivity	<---	Agility	1.000				
Adaptability	<---	Agility	1.381	0.231	5.978	***	Significant
Resilience	<---	Agility	1.005	0.177	5.690	***	Significant
Work readiness	<---	Agility	4.473	1.500	2.982	0.003	Significant

Model fit parameters; CMIN/DF = 2.263, GFI = 0.98, CFI = 0. 956, RMSEA = 0.071. Chi-square = 11.316, Degrees of freedom = 5, Probability level = 0.045. CFI: Comparative Fit Index, IFI: Incremental Fit Index, RMSEA: Root Mean Square Error of Approximation

## Discussion

Current study aimed to investigate the mediating role of nurses’ agility in the relationship between authentic leadership and work readiness among staff nurses. The current study revealed relatively high staff nurses’ agility and work readiness levels. Along the same vein, according to [51]. It was determined that subjects work readiness was relatively high with mean score of (296.37) which was at a medium-–high level. Concerning nurses’ perception of authentic leadership total mean scores, current study findings illustrated above-average mean scores; this is a good indicator the organization have to build upon and improve authenticity characteristics for both leaders and followers to enhance positive nurses work characteristics that in turn improve the quality of nursing care. Similarly [52], illustrated that the majority of nurses (98.4%) thought their leaders were exhibiting moderate to high levels of authentic leadership.

These findings can be interpreted in the light of the social support and social learning theories [21] both suggest that, when employees sense their leaders’ respect, consideration, concern, and support and perceive them as authentic, workers can more easily excel more effort [53] especially when authentic leadership combined with a personal energizing attribute as agility [15]. Social exchange theory [54] can also explain why individuals who perceive authentic leadership feel obligated to reciprocate with improved performance. The latter theory proposes that each person’s behavior is contingent on other individuals’ behavior. Subordinates working under an authentic leader’s guidance may feel, under the norm of reciprocity [55].

In the same context [23], revealed that studied nurses perceived their leaders as having above average level of authentic leadership. Also, this result was supported by [56] whose study stated that nurses reported their immediate supervisor‘s leadership behaviour to be moderately authentic.

Regarding correlations among authentic leadership, nurses, agility and work readiness; shows the presence of significant positive correlations between study variables. These findings could be explained as authentic leadership, such as moral integrity, care for others, and consistency between actions and moral principles, attempts to create a productive workplace [57]. Authentic leaders motivate and energize subordinates while bringing rich resources into the organization’s work environment.

Consistent with current study findings, an integrative review conducted by [58]. concluded that data from 40 studies three dimensions considered to be nurse manages impact supporting development of nurse work readiness: (a) creating a supportive environment by empowering graduate nurses, (b) supporting people by taking accountability for the supporting role and team characteristics, (c) and supporting learning by providing ‘on the job’ learning opportunities.

The findings of this study align with substantial empirical evidence [59], Based on a regression analysis, determinants of work readiness were positive school climate and leadership experience [60] & [61]. revealed the presence of positive correlation between authentic leadership and employee agility [62]. Explained that the relationship is logical since authentic leadership develops a sense of responsibility and respect among workers and fosters a positive and healthy work environment leading to higher productivity among staff.

Study findings indicate that the link between authentic leadership practices enhancing nurses work readiness is mediated by nurses’ agility. The mediation model suggests that authentic leadership improves nurses’ agility, which in turn enhances their work readiness. Without the development of agility, the positive effects of authentic leadership might not fully translate into work readiness. Agility acts as a crucial intermediary mechanism that facilitates this transformation. This mediating effect can be understood as authentic leadership theory indicates that authentic leadership not directly impact employees’ behaviors and attitudes rather intervening variables help to cultivate the authentic leadership influence by enhancing hope, trust, positive emotions, and optimism that in turn enhance nurses’ job performance. This interpretation justifies our moderated model that the direct impact of authentic leadership on work reading was not statistically significant but indirectly through nurses’ agility shows high statistically significant influence over nurses’ work readiness [20].

In line with the results of the present investigation [63], study shown that genuine leadership positively and statistically significantly impacted workers’ perceptions of their level of interpersonal trust and occupational self-efficacy. It’s important to note that other findings have been reported in the literature, though. For example [64], revealed that in order to determine if and to what extent internal moral viewpoint, self-awareness, relational transparency, balanced processing, and each of these components predicted technological preparation, multiple regression analysis was employed in their study.

Organizations can effectively provide nurses with measures that foster adaptation abilities with autonomy and support for taking proactive and adaptive measures in providing patient care beside improving leaders’ authentic characteristics as transparency, self-awareness, and balanced processing. These measures collectively according to current study analysis improve nurses’ work readiness that in turn enhance quality of care provided for patients.

To clarify, authentic leadership effect on nurses’ work readiness was insignificant according path analysis and the only significant effect of authentic leadership effect on nurses’ work readiness cultivated and fully mediated by nurses’ agility. So, organization have to pay more attention to enhancing agility mindset of nurses as attention paid for enhancing leadership abilities.

The results of this investigation are consistent with earlier research. Health organizations must become more nimble in order to assist clinical nurses’ career management, manager selection, and performance enhancement [65]. In a similar vein [41], discovered that the study’s data corroborated the theories positing a positive association between the evaluation of the clinical nurse’s work environment and nurse executive true leadership [52]. emphasized that in order to enhance nursing care quality and, by extension, patient outcomes, policy makers, administrators, and nursing leaders must manage and improve the work environment for nurses [66]. consistently concluded that nurses’ in-role performance is positively impacted by servant leadership. Although these findings contradict [67] finding that nurses’ resilience declined when.

### Limitations

Limitations were identified in this study; one of which is the cross-sectional design of the study. This method provides a moment-in-time view of the interactions between variables, but it is not suitable for determining causality or the potential evolution of these relationships. Additionally, the sample size although was sufficient for the study’s statistical analysis, a more diverse and extensive sample will help to draw generalizable conclusions. The geographical limitation of the study into one city also hinders generalizability of study findings.

## Conclusion

The present research add to existing nursing studies by working out the complex mechanisms underlying the relationship between authentic leadership and nurses’ work readiness. The current study’s findings indicate that nurses’ agility can fully mediate the relationship between authentic leadership and work readiness. authentic leadership has a significant indirect effect on work readiness, accounting for nurses’ agility as the cultivating variable that transmits authentic leadership effects to improve nurses’ work readiness. The path analysis revealed that the direct effect of authentic leadership on work readiness was insignificant, but the indirect effects, mediated through nurses’ agility, were significant. Therefore, nurses’ agility can be considered a full mediator in this model relationship.

### Future research recommendations

Building on the current findings, future research in this context should consider study limitations through:

Studies with a longitudinal design may shed light on how these connections change over time and how authentic leadership affects workers’ preparation for the workforce in the long run. To further generalize the results, it would be beneficial to look into the effects of authentic leadership in various healthcare settings and cultural contexts. Consider additional factors that could influence work readiness, such as job satisfaction, clinical placement or organizational culture. Insignificant direct relation between authentic leadership and work readiness needs more extensive analysis to find out the reasons and justifications.

### Implications in nursing practice and for policymakers

Organizations should commit to selecting authentic leaders open to nurses’ feedback and build a more transparent and value-based work environment. Organizations should enhance leaders’ authenticity by conducting training programs focused on developing authentic leadership qualities. Engage nurses in decision-making can enhance nurses’ agility the improve readiness. Nursing management should adopt policies that enhance delegation of authority and transparent feedback from nurses that also enhance nurses’ agility and in turn work readiness. Policymakers need to formulate promotion policies that take into consideration subordinates feedback regarding their leaders practices.

## Data Availability

The datasets generated and analyzed during the current study are available from the corresponding author on reasonable request.
